# CAD-EYE: An Automated System for Multi-Eye Disease Classification Using Feature Fusion with Deep Learning Models and Fluorescence Imaging for Enhanced Interpretability

**DOI:** 10.3390/diagnostics14232679

**Published:** 2024-11-27

**Authors:** Maimoona Khalid, Muhammad Zaheer Sajid, Ayman Youssef, Nauman Ali Khan, Muhammad Fareed Hamid, Fakhar Abbas

**Affiliations:** 1 Department of Computer Software Engineering, Military College of Signals, National University of Science and Technology, Islamabad 44000, Pakistan; mkhalid.msse22mcs@student.nust.edu.pk (M.K.); msajid.msse-27mcs@student.nust.edu.pk (M.Z.S.); nauman@mcs.edu.pk (N.A.K.); fareedhamid@hotmail.com (M.F.H.); 2 Department of Computers and Systems, Electronics Research Institute, Cairo 11843, Egypt; aymanmahgoub@eri.sci.eg; 3 Centre for Trusted Internet and Community, National University of Singapore, Singapore 119077, Singapore

**Keywords:** multi-eye disease, feature fusion, deep learning

## Abstract

**Background:** Diabetic retinopathy, hypertensive retinopathy, glaucoma, and contrast-related eye diseases are well-recognized conditions resulting from high blood pressure, rising blood glucose, and elevated eye pressure. Later-stage symptoms usually include patches of cotton wool, restricted veins in the optic nerve, and buildup of blood in the optic nerve. Severe consequences include damage of the visual nerve, and retinal artery obstruction, and possible blindness may result from these conditions. An early illness diagnosis is made easier by the use of deep learning models and artificial intelligence (AI). **Objectives:** This study introduces a novel methodology called CAD-EYE for classifying diabetic retinopathy, hypertensive retinopathy, glaucoma, and contrast-related eye issues. **Methods:** The proposed system combines the features extracted using two deep learning (DL) models (MobileNet and EfficientNet) using feature fusion to increase the diagnostic system efficiency. The system uses fluorescence imaging for increasing accuracy as an image processing algorithm. The algorithm is added to increase the interpretability and explainability of the CAD-EYE system. This algorithm was not used in such an application in the previous literature to the best of the authors’ knowledge. The study utilizes datasets sourced from reputable internet platforms to train the proposed system. **Results:** The system was trained on 65,871 fundus images from the collected datasets, achieving a 98% classification accuracy. A comparative analysis demonstrates that CAD-EYE surpasses cutting-edge models such as ResNet, GoogLeNet, VGGNet, InceptionV3, and Xception in terms of classification accuracy. A state-of-the-art comparison shows the superior performance of the model against previous work in the literature. **Conclusions:** These findings support the usefulness of CAD-EYE as a diagnosis tool that can help medical professionals diagnose an eye disease. However, this tool will not be replacing optometrists.

## 1. Introduction

The eye is considered a key organ that is essential for functioning vision. Illnesses affecting the eyes have the potential to cause irreparable damage to the retina, which might lead to vision impairment or even blindness.

These eye-related disorders pose obstacles to fundamental activities such as reading, recognizing faces, and driving. Consequently, vision impairment will have a great impact on a person’s quality of life. It is estimated that there is a minimum of 2.2 billion people struggling with various forms of visual impairment worldwide. The economic costs of vision impairment are significant; they are projected to be USD 411 billion globally per year. Statistics highlight a substantial disparity in treatment access. Globally, only 36% of individuals experiencing vision impairment from refractive errors and a mere 17% of those affected by cataracts have undergone necessary and suitable treatments. These small percentages prove the pressing requirement for enhanced global initiatives aimed at addressing and preventing visual impairments, with a focus on the potential positive impact on both society and the economy [1]. The early identification and detection of eye diseases such as glaucoma play a crucial role in preventing irreversible vision loss. This is very important as the condition can lead to permanent visual impairment. One disease that affects the eye is glaucoma. Traditional methods for glaucoma detection have often shown limited accuracy [2]. However, a novel approach has been developed, enabling faster and more effective disease detection by analyzing the characteristics of the optic disk in retinal images. An essential factor in glaucoma is intraocular pressure (IOP), analogous to blood pressure but specific to eye pressure, and can be used for the classification of this issue. Elevated IOP can cause damage to the optic nerve, resulting in symptoms such as blurred vision and eventual blindness over time [3,4,5]. Because manual processing of eye pictures takes a lot of time and is prone to errors depending on the examiner’s skill level, automated techniques for glaucoma identification are essential. These days, an analysis of 50 automated computerized retinal images has become a useful screening technique for detecting a range of eye conditions and hazards. Glaucoma manifests in two forms, chronic (called open-angle glaucoma) and acute (closed-angle glaucoma), both of which can increase intraocular pressure. Notably, during the early stages of glaucoma, symptoms cannot be noticed in patients, posing a challenge to early detection. Detecting glaucoma at its early stages is crucial to prevent progression to irreversible blindness. In recent years, digital retinal images have emerged as a valuable tool for conducting 57 glaucoma screenings. Various techniques and procedures have been developed to identify retinal abnormalities associated with glaucoma, enabling early detection and intervention for this vision-threatening condition. A non-invasive medical treatment called fundoscopy, often known as ophthalmoscopy, is used to look at the inner parts of the eye (retinal blood vessels, retina, etc.). This examination, which is usually carried out by medical specialists like ophthalmologists, internists, neurologists, or pediatricians, offers an in-depth examination of the eye’s central nerve system, arteries, and veins.

Its main function is to diagnose and track down a range of eye diseases, including glaucoma, retinal hemorrhages, diabetic retinopathy, and macular degeneration [6]. In fundoscopic examination, eye drops must be used. The patient’s pupils are dilated with these topical and short-acting eye drops. Following this, an ophthalmoscope, a specialized optical device, is employed to illuminate the retina through the pupil. This illumination enables the formation of an image of the retina, visible through the pupil, offering valuable insights into overall eye health and the presence of any abnormalities or diseases [6]. These images can be captured using a camera that is integrated into an ophthalmoscope that focuses on the retina or other eye structures [7].

The collected fundoscopic images serve as a valuable data source for the development of artificial intelligence (AI) systems designed to support various eye illnesses in terms of the diagnosis and treatment. Widely recognized open ophthalmological imaging datasets, including MESSIDOR, EyePACS, DRIVE, and E-ophtha, have been used by a large number of machine learning (ML) researchers in their research [8,9,10,11]. Another way to detect such diseases is AI systems, which can be used to recognize and evaluate patterns related to certain eye disorders by training AI models using a large dataset of fundoscopic pictures.

This saves time and improves accessibility to care. Also, AI systems have the ability to standardize treatment steps and enhance overall treatment outcomes for the patient.

Diabetic retinopathy is another eye issue that is a complication of the diabetes condition. Eye diseases are a major cause of blindness in elderly and middle-aged people because they affect the eyes. DR is considered a major global health problem [12]. According to estimates, the prevalence of vision-threatening diabetic retinopathy (VTDR) and diabetic retinopathy (DSR) in individuals with diabetes in the US is 4.4% and 28.5%, respectively [13]. Projections of diabetic retinopathy suggest that by 2045, DR and VTDR will affect around 160.50 million and 44.82 million people, respectively, globally. People living in the Middle East, North Africa, and Western Pacific will be more affected by these conditions [14]. Diabetic retinopathy can be identified by recognizing certain features in the retina. Clinical classification can classify the case to mild, moderate, and severe levels of diabetic retinopathy [12]. It has been demonstrated [15] that in countries with developed healthcare systems, like the United Kingdom, blindness can be effectively decreased through the early detection of vision-threatening conditions and timely treatment of them. Another exam that is used in the diagnosis of diabetic retinopathy is retinal photography. This screening method for diabetic retinopathy has proved superior performance in certain cases compared to an in-person eye exam [16]. Most low- and middle-income countries have difficulties in implementing systematic diabetic retinopathy screening programs. Hence, it is important to build cost-effective automated methods for screening and treatment of diabetic retinopathy [15]. Deep learning (DL) models are powerful machine learning models that have the capability to learn and extract important features from images. These models, when trained on a large dataset, can generalize for classification problems [10]. DL models are proven to have impressive results in implementing automated systems for an image analysis of eye images. In recent years, DL models were used widely for medical imaging classification, especially eye diseases. In [17], a deep learning-based convolutional neural network is proposed for the classification of eye diseases. In [18], a deep learning framework for retinal disease classification is proposed. All these research papers prove the importance of DL in medical imaging classification.

Hence, integrating deep neural networks into the screening process for diabetic retinopathy has importance. DL models can help with the classification of diabetic retinopathy lesions with high accuracy [15].

Another eye disease is called glaucoma. This disease can cause vision loss and blindness if left untreated. Globally, glaucoma is the primary cause of irreversible blindness and can affect the quality of a person’s life [19]. A class of progressive visual neuropathies known as glaucoma is defined by changes in the optic nerve head brought on by the degradation of retinal ganglion cells and retinal nerve fiber layers [20]. The condition is associated with damage to the optic nerve brought on by high intraocular pressure (IOP), which results in the degeneration of retinal ganglion cells [21]. Glaucoma is more commonly observed in adults. It is estimated that in 2020, over 76 million people were affected by glaucoma and it is anticipated that the number of adults affected by glaucoma will reach 111.8 million by 2040 [22]. The most common glaucoma type is primary open-angle glaucoma (POAG). This type is estimated to be affecting 2.2% globally. This means that approximately 57.5 million people are affected by it worldwide. In Europe, the POAG percentage is estimated to be 2.51%. This means that it affects around 7.8 million individuals.

Primary angle-closure glaucoma (PACCG) is another type of glaucoma, which is less common and affects only 0.17% of individuals younger than 40 years. Currently, the diagnosis of glaucoma needs eye examination and medical expertise, which might be costly or not available [23]. Furthermore, there is a significant amount of similarity in ocular characteristics between those without glaucoma and people who have the disease in its early stages [24,25].

Important instruments for the diagnosis of glaucoma include color fundus photography with red-free imaging, Heidelberg retinal tomography (HRT), and visual field (VF) tests [26]. In recent years, there is an increased interest in building AI systems for medical diagnoses, which can help with glaucoma screening and diagnoses. “DYSEO” is a web-based telemedicine program designed to test for glaucoma. The system combines Optical Coherence Tomography (OCT) and retinography in the detection of the glaucoma condition. The screening program accuracy was reported as medium to high [27]. The integration of artificial intelligence (AI) and machine learning (ML) algorithms into telemedicine screening programs can enhance the diagnostic accuracy. Much of the works in the literature related to glaucoma classification have faced challenges like a small number of images in datasets and lack of variations in images. All these challenges contribute to limiting the robustness and generalizability of developed models [28,29]. The creation of a model that can handle photos taken in a variety of environmental settings is essential to overcoming this limitation. In this work, a novel DL model that combines the features extracted from two of the most efficient DL models is proposed. The proposed model is built to classify four different eye diseases. A large dataset that is collected from different public and private sources is used to train the proposed model. The main objective of this robust model and huge dataset was enhancing the accuracy and reliability of retinal classification systems. The proposed system is evaluated against other DL models and other systems in the literature.

### 1.1. Research Motivation

**Figure 1:**
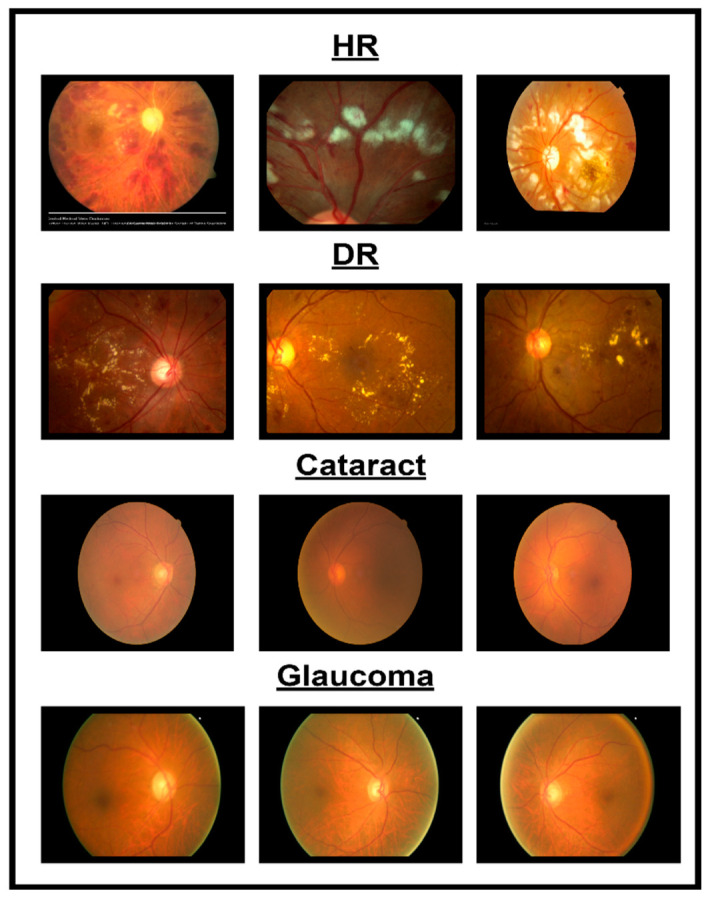
Illustration of eye disorders.

In spite of the progress in different methodologies for classifying eye diseases from digital images, encompassing normal cases, diabetic retinopathy, hypertensive retinopathy, glaucoma, and contrast-related conditions, substantial challenges persist. Figure 1 shows images of different eye disorders.

- Even with the utilization of sophisticated image processing technologies, accurately delineating features from images related to normal cases, diabetic retinopathy, hypertensive retinopathy, glaucoma, and contrast remains challenging. The challenges involved in precisely locating and extracting features associated with eye diseases contribute to this difficulty.
- A dataset combining photos from various eye illnesses such as hypertensive retinopathy, diabetic retinopathy, glaucoma, and contrast is not publicly available. Because of this, it is hard to implement an automated system to classify different eye diseases.

This study aims to develop an automatic system for the classification of different eye diseases. To achieve this goal, a first step is creating a dataset for four different diseases (diabetic retinopathy, hypertensive retinopathy, glaucoma, and contrast-related diseases) and a normal eye. The second step is to create a multi-layer deep learning (DL) model that can generalize to different eye diseases classes. The two steps combined produce a CAD-EYE system with multi-layered architecture. Through extensive training, this CAD-EYE system becomes proficient in classifying different eye diseases. Regarding the diseases, the analysis of the eye was performed by a skilled ophthalmologist.

### 1.2. Research Contribution

This work proposes a novel deep learning (DL) model to address the problem of recognizing four different eye diseases. Also, in this work, a new dataset was collected from reputable internet websites and a private collected dataset from previous research. The following steps summarize the significant contributions of the CAD-EYE system:

- In this work, the researchers compiled a substantial dataset consisting of 10,000 photos sourced from reputable internet platforms and supplemented by private datasets from previous studies. This large dataset was important since it enabled the model to achieve remarkably high classification accuracy.
- This work is the first to introduce a Fluorescence Imaging Simulation algorithm into multi-eye classification research. The image processing algorithm along with the proposed feature fusion model allowed the model to achieve higher accuracies than reported systems while classifying four different eye diseases.
- In this work, the feature fusion technique was incorporated to combine the features from two DL models to build the CAD-EYE system. The approach resulted in the creation of a multi-layered model that was successful in solving the classification problem.
- Additional layers (custom layers including dense layers following the feature fusion process, which help refine features extracted from both models) are added to the design of the CAD-EYE model to enable the model to classify a number of eye-related diseases. The convolutional neural network (CNN) models (MobileNet, EfficientNet) are used to extract features associated with eye disorders, and these features are subsequently combined through the feature fusion approach.
- The authors claim that this is the first effort to develop an automated system that is superior to current approaches in the identification of five different eye classes (normal, diabetic retinopathy, hypertensive retinopathy, glaucoma, and contrast-related eye disorders), as illustrated in Figure 1.
- Our systems exhibited superior performance compared to the approaches proposed in the available research, achieving a remarkably higher accuracy percentage of 98%.

### 1.3. Paper Organization

The structure of this paper is as follows: Section 2 includes the literature survey. Section 3 outlines the proposed model design. Section 4 presents the experimental results. In Section 5, our findings are compared with those of contemporary studies in the field. Section 6 conducts a comprehensive discussion of the research findings. Finally, Section 7 presents the study’s conclusions.

## 2. Literature Survey

Much of the papers in the literature include the extraction of blood vessels or the partitioned location of lesions, and the instruments and strategies utilized are different, contributing to the complexity of framework design [30,31].

Daniel Shu and Wei Ting et al. emphasize that deep learning has gained considerable attention in recent years [32]. Despite that deep learning models were invented a long time ago, their impact on picture identification, natural language processing, and speech recognition started to appear mainly in the healthcare industry [33,34]. The application of deep learning (DL) in ophthalmology, specifically in fundus pictures, has facilitated the classification of conditions such as a glaucoma-like disk, macular edema, and age-related macular degeneration. In primary care and community settings, DL in ocular imaging and telemedicine enables the screening, identification, and follow-up of patients with serious eye problems [35,36]. However, there are still some challenges in using deep learning models in ophthalmology. These challenges include problems with clinical implementation, difficulty explaining the results for doctors and patients, and difficulties convincing doctors and patients to receive a diagnose from “black-box” models.

Ophthalmologists’ manual diagnosis of DR retina fundus images is expensive, time-consuming, and prone to errors. Deep learning, particularly convolutional neural networks, has shown enhanced performance in the classification of medical images, offering a promising avenue for the diagnosis of DR and other medical conditions [37]. In research paper [38], novel deep learning models for diabetic retinopathy classification are investigated and examined. The analysis also includes an assessment of datasets specific to DR in color fundus retinas. Additionally, certain complex challenges have been identified, calling for further research. The proposed deep learning approach significantly enhances the detection of the disk and cup. However, because medium-sized cups were so common in the model’s training, it has a tendency to overestimate tiny cups and underestimate large ones. The conventional approach to glaucoma identification is measuring increased intraocular pressure (IOP). In an earlier work [39], binary distinctions between participants with and without glaucoma were made using features that were calculated at the image level utilizing picture attributes. Three major layers are commonly seen in CNN designs, or convolutional neural networks. Numerous research studies have explored the identification of eye diseases, employing diverse methodologies and technologies crucial for enhancing the precision and efficiency of disease diagnoses in the field of ophthalmology. In a research paper published by Malik et al. [40], the emphasis was on applying artificial intelligence techniques in healthcare systems, particularly for fast diagnoses. They made use of machine learning models including a Random Forest, a decision tree, Naïve Bayes, and a neural network. These models were trained with varied data, including patient information, age, disease history, and clinical observations. In the work, it was found that Random Forest and decision tree algorithms demonstrated high accuracy rates that were better in comparison to previous research papers.

In [41], the researchers investigated the development of computer-aided diagnostic (CAD) systems for glaucoma detection. In the study, a convolutional neural network (CNN) was used to extract important features from retinal images. In the work, the objective was a binary classification between glaucoma and non-glaucoma using a dataset, utilizing and comprising 1200 retinal images. The model achieved a high accuracy of 99%, proving its ability to enhance glaucoma diagnoses. In [42], the authors built a model to differentiate individuals with eye disease and healthy people. The authors were able to train a model on datasets containing both patient and healthy individuals’ retinal fundus images. The model results in high accuracy ranging from 96.5% to 99.7%, proving the effectiveness of their approach. In [43], authors addressed the critical problem of retinal disease, concentrating on the early diagnosis of diseases. The authors of the work used deep learning models incorporating CNN-based models. These models were used to classify different types of eye disorders. The model achieved average accuracy ranging from 81% to 94%, along with an average F1-score of 0.96 for normal retinas. Another work [44] focused on diabetic eye disease (DED). The authors applied CNN methods for the classification of retinal eye diseases. The proposed model is trained to classify a range of DED classes; also, an ophthalmologist carefully examined a large dataset of retinal fundus images to verify the model. The overall accuracy (ACC) reached 81.33%, accompanied by 100% sensitivity and 100% specificity for multiclass classification, showcasing the potential of CNNs in detecting a range of diabetic eye diseases. Umer et al. [45] investigated the application of Optical Coherence Tomography (OCT) in the computer-aided detection and classification of retinal eye disorders. Currently, ophthalmologists rely on manually examining OCT images, a process prone to inaccuracies and subjectivity. To address this, the study introduced various methods to automate disease detection, making use of a four-class retinal eye disorder dataset that was made available to the public. To extract deep feature vectors, modified versions of the AlexNet and ResNet-50 models were used. With a remarkable overall average accuracy (ACC) rating of over 99.95%, the suggested strategy for identifying retinal eye illnesses demonstrated the potential for accurate and objective disease detection. Gargeya et al. [46] addressed the computer-aided diagnosis of diabetic retinopathy (DR) using image processing techniques and CNN models. Their study involved a substantial dataset comprising 75,137 available retinal eye images from different patients. The assessment of model performance uses the area under the receiver operating characteristic curve (AUC) as an evaluation metric, employing five-fold cross-validation. Notably, the model’s AUC for diagnosing DR was 97%, while its sensitivity and specificity were 94% and 98%, respectively, highlighting its effectiveness in automated disease diagnoses. In summary, these studies collectively showcase diverse approaches and technologies in the field of ophthalmology for detecting and diagnosing a wide spectrum of eye diseases. They illustrated how automated diagnostic technologies, deep learning, and artificial intelligence might improve illness diagnoses’ efficiency, objectivity, and accuracy. The authors of [47] tackle the crucial challenge of detecting and classifying diabetic retinopathy (DR), a significant concern for diabetic patients vulnerable to severe visual impairment. They introduce an innovative automated system named DR-NASNet, which employs advanced techniques, including preprocessing methods such as Ben Graham and CLAHE, data augmentation to address class imbalance, and the combination of dense blocks within the NASNet architecture. In addition to achieving impressively accurate state-of-the-art outcomes, the system keeps its model size small and complexity low. Through the utilization of combined datasets and a linear SVM classifier, DR-NASNet proficiently categorizes DR images into five severity levels. This breakthrough helps build automatic systems to help ophthalmologists and offers an efficient tool for the early classification of DR. This helps the early detection of eye diseases and to prevent vision loss. Study [48] also has a nice contribution in the early detection of hypertensive retinopathy (HR). In the work, authors introduced the Incept-HR system combined with the collection of the Pak-HR dataset, providing a new contribution to the application of deep learning models in healthcare. The Incept-HR system proved its efficiency and impressive performance, making it an important diagnostic tool. The proposed model was compared with established models like VGG19 and VGG16 and outperformed their performance. The work is considered a step forward in achieving more eye disease classification models. On the other hand, Ref. [49] introduced a novel diagnostic model for hypertensive retinopathy classification called “Mobile-HR”. The model is based on fine tuning a MobileNet model using transfer learning. HR is a critical eye disease caused due to elevated blood pressure, resulting in various visual symptoms. Experimental results prove the effectiveness of the model in classifying HR disease in eye images.

The classification and investigation of cataracts necessitate a comprehensive understanding of their diverse manifestations within the eye lens. Both subjective observation and objective measurement techniques continue to be widely employed in this field. Notably, techniques such as a Scheimpflug slit image analysis, among the latter, offer a more precise means of identifying early transparency breakdowns. Objective approaches are pivotal in epidemiological research as they facilitate the accurate monitoring of risk variables, including UV-B radiation exposure, and their potential role in cataract development. This significance arises from the fact that age-related changes in lens transparency occur before apparent opacifications. Longitudinal cohort studies, involving repeated examinations, are imperative for gaining deeper insights into the multifactorial processes associated with cataracts. Subjective evaluations alone may fall short in detecting minor changes in transparency. In summary, objective techniques for categorizing cataracts are indispensable for advancing our understanding of this vision-impairing disorder and the linked risk factors [50]. As a substantial contributor to visual impairment and a critical public health issue, cataracts are addressed through an automated identification method utilizing retinal image categorization rooted in computer science. Recognizing the crucial role of an early diagnosis in preventing blindness, our approach involves employing deep learning networks to extract distinctive characteristics from infected images, coupled with preprocessing using the maximum entropy approach. Subsequently, the automated identification of four classes of cataract photos—normal, mild, medium, and severe—is carried out using conventional classification techniques, specifically SVM and Softmax. Noteworthy in our findings is the demonstrated efficacy and utility of our study, emphasizing that the features obtained through deep learning and categorized by Softmax exhibit superior accuracy. Our overarching objective is to enhance the prospects for timely intervention and improved visual outcomes by advancing the early detection of cataracts through the fusion of computer science and medical imaging [51]. The healthcare challenge presented by uveitis, with its potential for blindness and societal impact, has become more addressable with advancements in machine learning technologies. Through our extensive examination, encompassing detection, screening, and the standardization of uveitis nomenclature, we have identified prospective roles for AI in uveitis research. It is crucial to acknowledge, however, that the current state of AI in uveitis diagnoses faces challenges, including overall subpar model performance, limited datasets, insufficient validation studies, and a lack of publicly available data and codes. Despite these obstacles, artificial intelligence holds significant potential in identifying and diagnosing ocular abnormalities associated with uveitis. Further research endeavors are essential, along with the creation of large, representative datasets for training and validation, to unlock its full potential and ensure its reliability and fairness [52]. From this literature survey, it is clear that there is a research gap in the area of the classification of eye diseases. In this work, a novel model is proposed for multi-eye disease classification. Also, a new dataset combined from previous datasets is introduced. This work is considered one step towards filling the research gap explored in this literature survey. Table 1 shows an overview of previous research articles related to the classification of eye illnesses.

## 3. Proposed Approach

**Figure 2:**
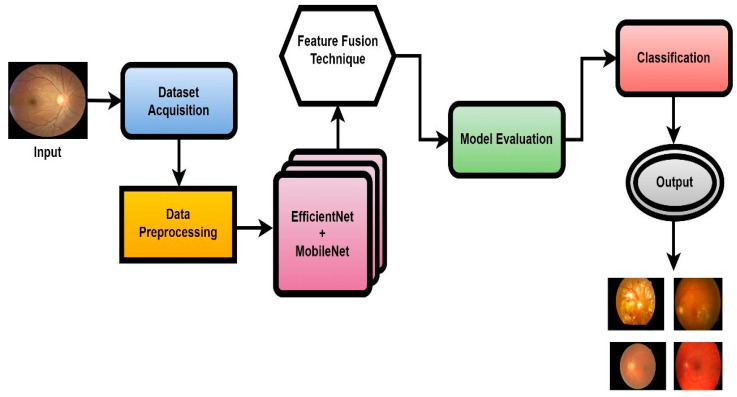
The systematic flow chart of the CAD-EYE system of eye disease classification.

In this work, a novel system named CAD-EYE, combining the strengths of EfficientNet and MobileNet, is introduced. The CAD-EYE approach is deployed to categorize images of eye diseases, discerning between diabetic retinopathy, hypertensive retinopathy, glaucoma, contrast-related issues, and normal cases. Within the CAD-EYE system, the feature fusion methodology of EfficientNet and MobileNet is utilized to extract valuable features, employing transfer learning for training on eye-related abnormalities. The CAD-EYE system incorporates essential mechanisms for detecting images depicting eye diseases and recognizing the mentioned issues. The system steps are presented in Figure 2. Throughout the training phase, the parameters are continuously refined by fusing the characteristics from EfficientNet and MobileNet. To combine features using element-wise multiplication, a feature transform layer is added. Lastly, the application of an XGBoost classifier increases the classification results.

A noteworthy advancement in the areas of machine learning and computer vision is the creation of the CAD-EYE model. This inventive methodology leverages the capabilities of two well-established architectural paradigms, EfficientNet and MobileNet, culminating in a unique hybrid model with the potential to surpass its predecessors. The proposed model combines the effective feature extraction of EfficientNet with the strong training stability and transferability of MobileNet; this combination has the potential to increase the model performance and can be applied to a variety of applications. This model can be adapted to different applications; this is considered as a distinctive feature of the model. Another advantage of this model is its ability to work in different applications with medium resources, making it suitable for limited-resource applications. This proposed model is used in different applications and proved superior performance over traditional methods. The feature extraction of both models is enhanced because components such as MB-Conv layers and SE blocks in EfficientNet are combined with inverted residual blocks in MobileNet.

### 3.1. Data Collection and Preprocessing

**Figure 3:**
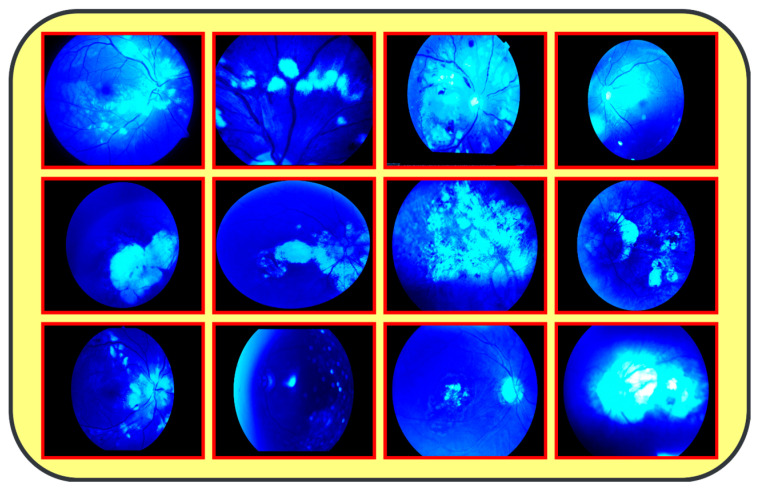
Results from image processing algorithm.

The proposed model (CAD-EYE) was trained and tested using the Multiple_EYE dataset that was collected especially for this work. This dataset contains 65,871 photos. These images were collected from different private and public sources. The sources included internet platforms and ophthalmic treatment facilities. In the collection processes, the Maestro2 OCT-Fundus Camera system is used, which is a user-friendly device that performs automatic alignment, focus, and capture with a single touch. This system enables a detailed analysis of the macula, optic disk, and anterior segment, with reports that can be automatically exported, printed, or integrated with EMR systems in common file formats. Explicit consent to use the data in research activities was acquired from patients and their doctors, with a promise of confidentiality of the data. This ensures accessibility of the data for other researchers while protecting the patients’ privacy. The collected dataset (Multiple-Eye) contains eye images pertaining to different eye conditions like diabetic retinopathy, hypertensive retinopathy, glaucoma, and contrast, and normal images. The training dataset was divided into normal class and eye disease photos by qualified ophthalmologists. The ophthalmologists’ experience in detecting eye-related traits was quite helpful in dividing the dataset. It used the Fluorescence Imaging Simulation preprocessing approach to enhance the image and make the feature extraction process more accurate. The technique strengthens the green channel of the image, imitating fluorescence, which is critical in some pathologies of eye fundus images. Fluorescence Imaging Simulation increases the visibility of these regions to clearly explain where the model is looking while deciding. More generally, the integration of the Fluorescence Imaging Simulation technique reflects a continued effort to increase transparency and thus reliability of deep learning models for improved accessibility and trustworthiness in numerous applications, as shown in Figure 3.

Figure 1: A detailed breakdown of the 65,871 eye photos involved in the research study. Table 3: Three datasets used to create the training and testing sets of fundus images, where different configurations are involved for every dimension. All images used in the experiments were uniformly resized to 700 × 600 pixels with their corresponding binary labels generated. This image size was chosen to balance the level of details needed from each image and the computational cost needed by deep learning models. In total, there were 65,871 photos within the dataset, and 9393 of the photos were used in order to evaluate the system. To make the dataset fair, proper classification into different classes was performed, and the number of images in each class before and after the onset of disease was balanced. The photos were preprocessed before feeding into the algorithm devised for the CAD-EYE model, which included resizing to 700 × 600 pixels. Standardization was further used to reduce variation across data points. The CAD-EYE system was trained and tested with the help of the dataset called Multiple-EYE, including images that were originally saved at a resolution of 1125 × 1264 pixels. Table 2 effectively simulates fluorescence in eye fundus images, outlining areas of interest and hence improving the interpretability of medical imaging. The images, sourced from three distinct origins, were resized to standard dimensions of 700 × 600 pixels for the purpose of the simplification and standardization of the dataset. To enhance image features and eliminate interference, the Grad-Cam technique was applied to preprocess the images, as depicted in Figure 3. Employing Grad-Cam on eye fundus images facilitated the identification of important areas and their importance in identifying the presence of eye diseases. This method helped identify distinguishing features that had a major impact on CNN’s predictions on the diagnosis of glaucoma, diabetic retinopathy, hypertensive retinopathy, and contrast images.

### 3.2. Data Augmentation

It is clear from Table 3 that there is a biased distribution of data. This bias will cause the classification model to favor a certain class over others. Investigating different approaches for balancing the data is critical to achieve more generalized models. To address this challenge, usually “data augmentation” techniques can be applied on the data to balance the data. Data augmentation generates extra data points from existing data, hence enhancing the dataset’s diversity. Alongside a data augmentation technique, other techniques may be applied to solve the data balancing problem. These techniques include resampling methods like oversampling and undersampling. Also, other procedures can be applied like boosting, bagging, or GANs. Each of these procedures has its own advantages and can be tuned to the specific application. Also, other techniques like bagging, boosting, and GANs may be considered. Each of these techniques has its own advantages and can be tuned to the specific dataset used. Data augmentation is usually performed using a simple algorithm that does not require a deep learning mode or tuning of the hyperparameters. This algorithm or procedure involves different transformation steps that include rotation, flipping, and cropping in order to create new samples to be added to the training dataset. The data augmentation procedure usually creates balanced classes with no majority and minority classes. In this work, we apply data augmentation to the training dataset only; this is to enhance the model generalization without affecting the evaluation of the model.

By using the AutoAugment approach to automatically enhance b images during the training phase, DL models can be made more generic. The first step in this AutoAugment technique is creating the transformation pipeline and choosing the transformation policy. This transformation pipeline includes image resizing, horizontal flipping, and the chosen AutoAugment policy. The next step in this AutoAugment technique is loading the dataset that is organized in another directory. The Dataloader is used to load the dataset that needs augmentation. On the other hand, the ImageFolder class is used to apply the AutoAugment transformation to each image. The DataLoader is used during training to provide batches of augmented photos from the custom dataset. This step increases the performance of the model. The steps of the Auto Augmentation algorithm are shown in Table 4.

## 4. Proposed Architecture

**Figure 4:**
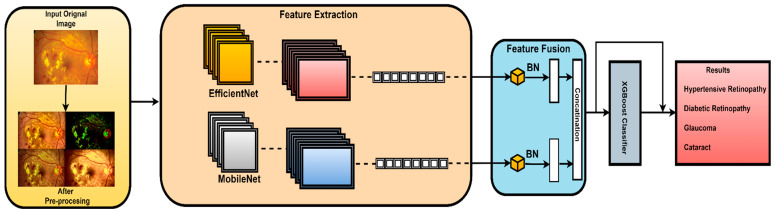
Architecture of the CAD-EYE system.

The proposed architecture combines the advantages of MobileNetV2 and EfficientNet using a well-designed feature fusion process. This leads to a final layer that is compatible with the XGBoost classifier. This means that the pretrained convolutional neural networks (MobilNetV2 and EfficientNet), which are known for their advantageous characteristics, serve as components in this novel model. In this study, features were extracted from the final convolutional layer of both MobileNetV2 and EfficientNetB0 models. This choice was made because the final convolutional layer provides high-level feature representations that are well suited for the classification task. The proposed fusion methodology extracts and harmonizes features from both architectures. This fusion methodology combines global average pooling and concatenation. In this work, element-wise summation was used to combine features extracted from both models as it effectively combines features while preserving spatial consistency. The subsequent dense layers further process the features and extract relationships. In this work, all features that were extracted from the final convolutional layer of MobileNetV2 and EfficientNetB0 were utilized as is, without additional feature ranking or selection. This is carried out to preserve all features to contribute to the classification accuracy. The final element in the architecture is the XGBoost classifier, which extends the model beyond CNNs. The proposed hybrid architecture combines deep learning models, offering a sophisticated ensemble approach for image classification tasks. The model benefits from combining the advantages of both deep learning models and high accuracy of the XGBoost model to achieve accurate classification results as illustrated in Figure 4.

Let M represent the MobileNetV2 model, and E represent the EfficientNet model.

*MobileNetV2 features : X_M_ = M(Input)* (1)

*EfficientNetB0 features : X_E_ = E(Input)* (2)

Make both models’ layers non-trainable.

(3) $$X_{M}^{\mathrm{fiozen}\;}=Freeze\left(X_{M}\right)$$

(4) $$X_{E}^{f_{\mathrm{rozen}}}=Freeze\left(X_{E}\right)$$

Let Dense () stand in for a dense layer and GlobalAveragePooling2D() for the global average pooling technique.

(5) $$X_{M}^{Pooled}=\;\mathrm{Global}\;\mathrm{AveragePooling}2D(X_{M}^{frozen})$$

(6) $$\;X_{E}^{Pooled}=\;\mathrm{Global}\;\mathrm{AveragePooling}2D(X_{E}^{frozen})$$

(7) $$X_{concat}=\mathrm{Concatenate}\;(X_{M}^{Pooled},\;X_{M}^{Pooled})$$

*X_dense1_* = *Dense* (128, *activation* = ‘*relu*’) (*X_Concat_*) (8)

*X_dense2_* = *Dense*
*(num_classes_, activation* = ‘*softmax*’) (*X_dense1_*) (9)

*f usion_model_* = *Model* (*inputs* = *[M.input, E.input], outputs = X_dense2_*) (10)

The proposed model architecture starts by using the pretrained MobileNetV2 and EfficientNet models, respectively, to extract features from the input images. After that, in order to preserve their learnt weights, all layers in both models are made non-trainable. The frozen features are then subjected to global average pooling, yielding X_MPˆooled and X_EPˆooled.

**Figure 5:**
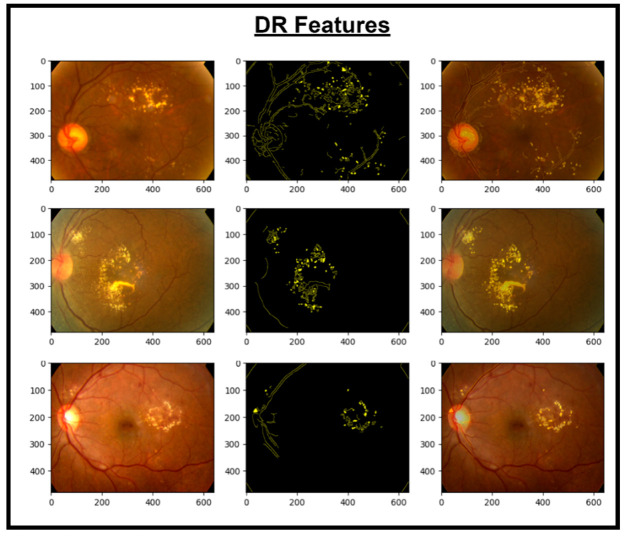
Images and DR extracted features.

**Figure 6:**
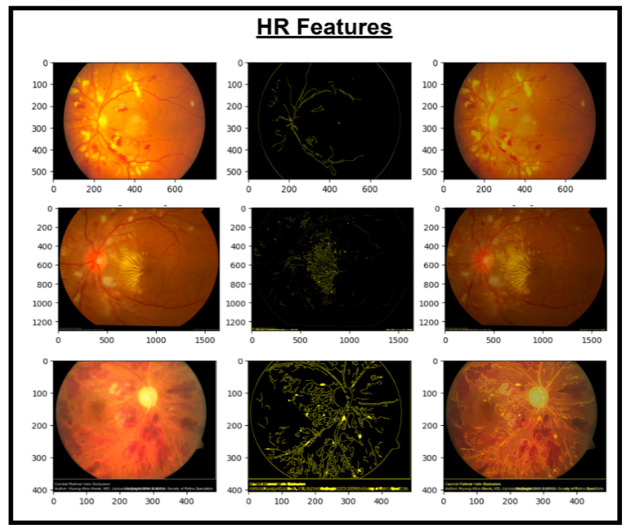
Images and HR extracted features.

**Figure 7:**
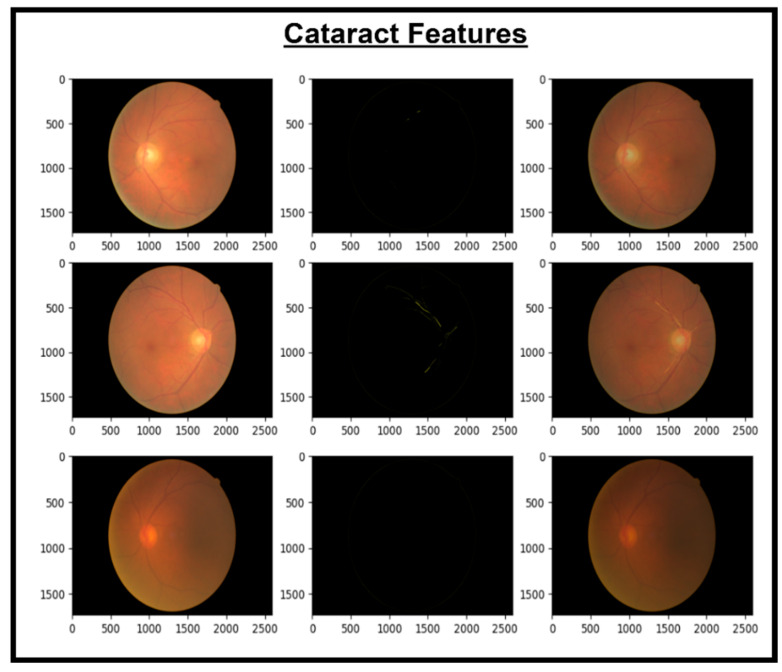
Images and cataract extracted features.

**Figure 8:**
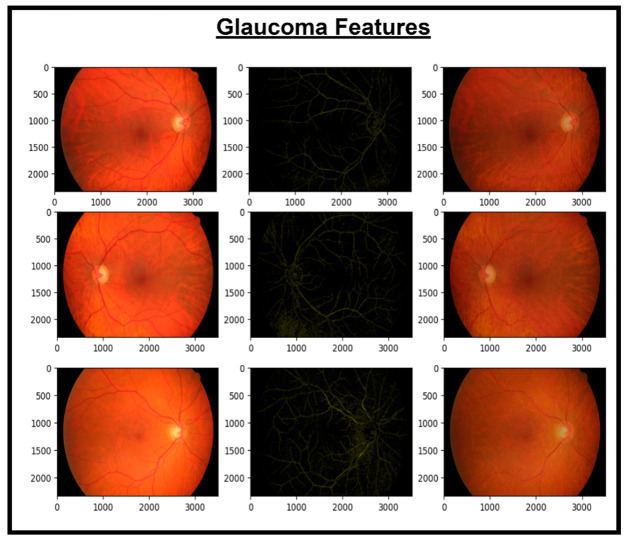
Images and glaucoma extracted features.

These features are then concatenated to form X_Concat. This vector uses, as a base, a dense layer with rectified linear unit (ReLU) activation, producing two new vectors, X_dense1 and X_dense2. Finally, the fusion model is defined with two inputs (from MobileNetV2 and EfficientNet) and one X_dense2. Subsequent to model definition, the architecture uses Adam as an optimizer and categorical cross-entropy loss. The model uses the SoftMax algorithm to transform output scores into probabilities; this is essential for the XGBoost model to work effectively. The architecture also uses accuracy as the monitoring metric. The fusion model is fitted to the training dataset for a specified number of epochs. Training yields a finely tuned model that incorporates the advantages of both MobileNetV2 and EfficientNet for image classification tasks. Figure 5, Figure 6, Figure 7 and Figure 8 show the infection images of the CAD-EYE system. Algorithm of the model is presented in Table 5.

## 5. Recognition of Eye Diseases

In an effort to automate the challenging process of diagnosing eye conditions using fundus photos, our novel fusion system, which we named CAD-EYE-Fusion, cleverly combines the key features of MobileNetV2 and EfficientNetB0. Illustrated in Figure 4, this creative design combines layers like global average pooling, dense, and batch normalization layers while skillfully combining features from both models using a mathematical fusion mechanism. Expertly designed skip connections accelerate network learning. The comprehensive CAD-EYE-Fusion system greatly improves image classification performance for multi-eye diseases by leveraging the integrated feature representation. The architecture boasts multiple dense blocks, constructed with depth-wise convolutional layers, max pooling, ReLU activation, and batch normalization, forming the core of CAD-EYE-Fusion. These dense blocks have skip connections within them that make training and connecting more effective. Comprising a total of three dense blocks, our model ensures consistency in input and output sizes, enabling effective feature learning. An extra layer—which includes layers like dense and batch normalization layers—that customizes the classification procedure provides the final classification outcome while preserving 850 nerve cells for peak performance. As a preprocessing step, batch normalization layers are smoothly integrated into CAD-EYE-Fusion, improving the model’s training and convergence. The detailed incorporation of batch normalization is provided in Table 6, emphasizing its significance in the comprehensive fusion system designed for robust eye disease classification from medical images.

*B* = {*X*_1···*m*_}, *γ*, *β* (11)

*B* = {*y_i_* = *BN_γ_*_∗_*_β_*(*X_i_*)} (12)

(13) $$\mu_{B}=\frac{1}{m}\overset{m}{\underset{i=1}{\sum }}X_{i}$$

(14) $$\sigma_{B}\leftarrow -\frac{1}{m}\overset{m}{\underset{i=1}{\sum }}X_{i}-\mu_{B}$$

(15) $$\;X_{i}-\frac{X_{i}-\mu B}{\sigma_{B}^{2}+\in }$$

## 6. XGBoost Classifier

In this study, the effectiveness of the XGBoost algorithm for the task of identifying features linked to glaucoma, hypertensive retinopathy, diabetic retinopathy, and contrast is investigated. The reason for using XGBoost was its superior performance in handling complex, high-dimensional data, which aligns well with the feature-rich representations extracted from the MobileNetV2 and EfficientNetB0 models. The algorithm, outlined in detail as Table 7 employs a gradient boosting framework with decision trees as base learners. In this work, the XGBoost model was customized through hyperparameters like the learning rate (*η*), regularization term (*lambda*), and the number of trees (*τ*) because it is especially well suited for binary classification jobs. For the feature extraction process in the realm of computer vision, we adopted depth-wise Conv2D, providing a more specialized convolutional operation. This modification was added to improve the model’s capacity in extracting complex patterns in retinal images.

During the training phase, decision trees are built iteratively, and the model is updated by updating the computed gradients (gi) and Hessians (hi) in every training sample. Additionally, the t-th tree prediction is gradually introduced to the ensemble throughout the model training phase of the XGBoost model, with the learning rate and weights established by the optimization process serving as a guide. All of the ensemble’s trees’ combined predictions make up the final prediction for a test sample, resulting in a robust recognition model. Mathematically, an objective function that balances a loss term and a regularization term is optimized by the XGBoost algorithm. The output for a testing sample (Atest) is computed as the sum of the predictions from each tree, weighted by the learning rate and tree weights. By using an ensemble technique, XGBoost is able to identify intricate relationships within the data and produce a dependable identification result for the categorization of diabetic retinopathy, hypertensive retinopathy, glaucoma, and contrast samples.

## 7. Results

A dataset comprising 65,871 fundus images, encompassing high-resolution normal, diabetic retinopathy, hypertensive retinopathy, glaucoma, and contrast images, was used to train the CAD-EYE system. These images of eyes have been gathered from a number of trustworthy websites. All 65,871 photos were scaled to 700 × 600 pixels in order to facilitate feature extraction and classification tasks. The CAD-EYE model was trained for 100 epochs. The proposed model evaluation metrics were calculated using a statistical analysis. The proposed model achieved an outstanding F1-score of 0.99. These measured metrics were used to compare the model with other models from the literature. The model was trained and developed on a PC equipped with 2 GB Gigabyte NVIDIA GPU 8 cores.

### 7.1. Experiment 1

**Figure 9:**
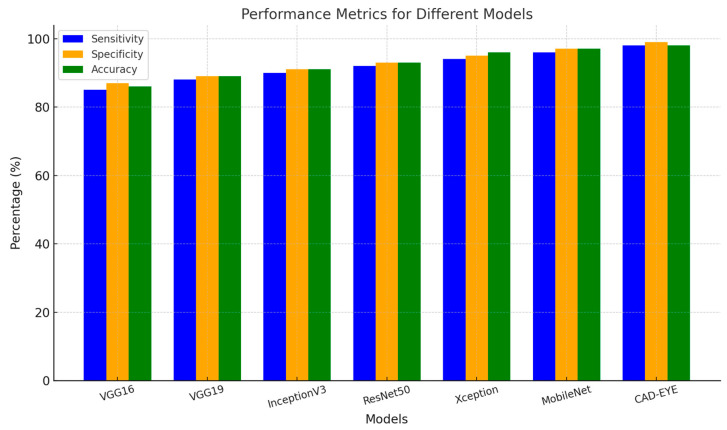
Comparison between some DL models and CAD-EYE.

In the first experiment implemented to test the proposed DL model, a comparison is made between the proposed model and other deep learning models like DenseNet-169, InceptionV3, VGG16, and MobileNet. All models were trained for an equal number of epochs. Comparative findings for accuracy, specificity, sensitivity, and F1-score between the CAD-EYE system and the MobileNet, VGG16, VGG19, Xception, InceptionV3, and ResNet models are shown in Table 8. The outcomes show that the CAD-EYE has better performance than other DL models. Figure 9 shows a graph that compares various DL models with the CAD-EYE model.

### 7.2. Experiment 2

**Figure 10:**
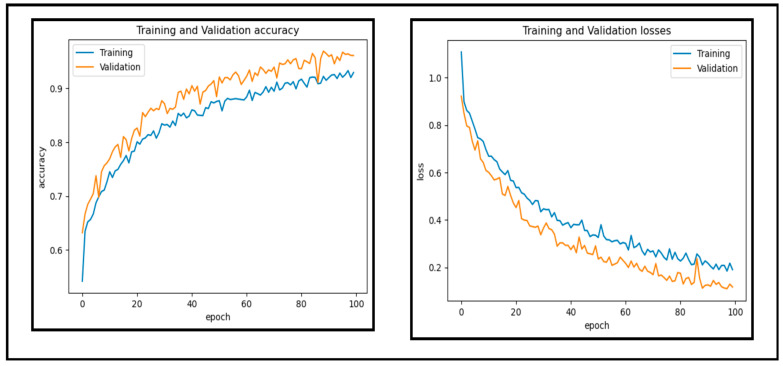
Accuracy and loss versus epoch for training and validation data.

**Figure 11:**
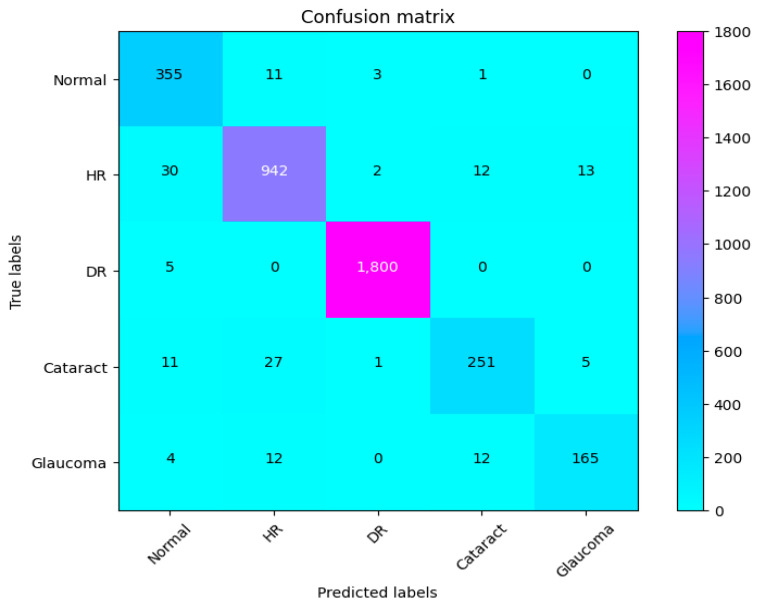
Confusion matrix of CAD-EYE.

In this experiment, a dataset of 65,871 fundus images obtained from different reputable online sources is used to test the performance of our proposed CAD-EYE system. Figure 10 and Figure 11 show accuracy and loss versus the epoch for the CAD-EYE model using training and validation datasets. The results demonstrate the high efficacy of CAD-EYE in both training and validation scenarios.

### 7.3. Experiment 3

**Figure 12:**
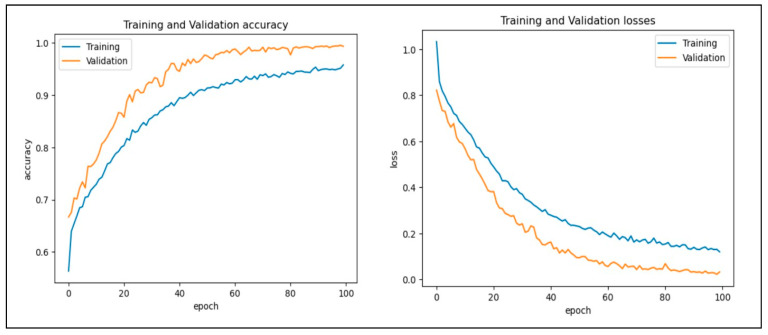
Accuracy and loss versus epoch for training and validation data using EDC dataset.

In this work, we assessed the efficiency of our proposed CAD-EYE technique using the eye disease classification (EDC) dataset [56] that was investigated. First, we looked at the loss function and analyzed how well the model performed using the EDC dataset on the training and validation sets. The training and validation accuracy of the CAD-EYE model trained on this dataset can be seen in Figure 12. Results show how exceptionally successful our model is in both training and validation scenarios. Furthermore, by utilizing the EDC dataset outlined in Table 9, we achieved a remarkable 99% accuracy on the validation and training datasets.

## 8. State-of-the-Art Comparison

Only a few studies investigated using deep learning algorithms to diagnose eye conditions using retinal pictures. Among these, the EDC research [58] stands out for utilizing deep learning with a small dataset to detect normal, cataract, glaucoma, and diabetic retinopathy cases in retinal images. The latest deep learning model addressing normal, cataract, glaucoma, and diabetic retinopathy detection is referred to as EDC [58]. In contrast, the CAD-EYE system we developed demonstrated outstanding outcomes, achieving values of 99.50%, 99.68%, 99.98%, 99.95%, 99.98%, and 1.0 for SE, SP, F1-score, Recall, and ACC, respectively. In EDC, the author of [58] noted that they utilized a very limited set of input fundus images for training, resulting in high precision and accuracy. However, it is crucial to mention that their dataset lacked approval from expert optometrists. In our case, the CAD-EYE system underwent testing and training on a dataset of 65,871 images, which received validation from expert optometrists. Consequently, we achieved a classification accuracy of 98%, representing significant advancement above the state-of-the-art work that is currently available. Table 10 presents a detailed performance comparison between the proposed model CAD-EYE and EDC model.

## 9. Discussion

The eye is a crucial element for a person’s work and activity. There are many illnesses that have an impact on how effectively the eyes work. Effective treatment of these diseases depends on early discovery. This article proposes a system for classifying various eye illnesses. The four different illnesses that the proposed model can identify in eye images are diabetic retinopathy, hypertensive retinopathy, glaucoma, and contrast-related eye diseases. This work combines different innovative ideas to achieve the goal of the classification of four different eye diseases. This work is the first to propose the use of a fluorescence imaging algorithm in multi-eye disease classifications. This image processing algorithm highlights important areas. The regions that are critical for classification are the focus of this image processing approach. Secondly, the authors suggest combining features extracted from two of the cutting-edge models to create a new feature vector. Also, a dataset is produced by merging several datasets obtained from different internet resources. This step is necessary since the model was trained on classifying four different diseases. The proposed model undergoes three different experiments to prove its superior performance. The first experiment is performed to evaluate the suggested model’s performance against other cutting-edge models (VGG16, VGG19, InceptionV3, ResNet, Xception, MobileNet). The presented results show the superior performance of the proposed method in comparison to these models. The results show that CAD-EYE was able to achieve 97% accuracy, which is higher than the best other models by 10%. In reviewing the confusion matrix in Figure 11, we observe that the model achieves higher accuracy for diabetic retinopathy (DR) cases, while normal eye images are occasionally misclassified. This variation in performance may stem from the distinct features associated with DR, which are more easily identifiable by the model compared to the subtle variations in normal eyes. The features of a normal eye may overlap with mild signs of disease or noise, leading to occasional misclassification. The second experiment conducted in this research is testing our proposed model using a dataset created by combining images from different online sources. The dataset contains 65,871 fundus images. The dataset contains high-resolution normal and diabetic retinopathy, hypertensive retinopathy, glaucoma, and contrast eye disorder images. The findings demonstrate that the suggested model can achieve accuracy levels greater than 95%. The third experiment conducted in this research is testing our proposed model against the EDC (Kaggle’s eye disease classification) dataset. It is noted that the EDC dataset contains a very limited set of input fundus images for training. However, it is crucial to mention that the EDC dataset lacked approval from expert optometrists. Our collected dataset for the CAD-EYE system consists of 65,871 images, which received validation from expert optometrists. The experiment is necessary to evaluate the model against similar work from the literature. The enhanced performance of the suggested system is demonstrated by a state-of-the-art comparison with a comparable work from the literature. Exceeding published accuracies in the literature, the suggested approach managed to attain better accuracy levels. In summary, the classification strategy for eye illnesses presented in this study is based on the fusion of characteristics and deep feature extraction. To the best of the authors’ knowledge, this is the first attempt to develop an automated system for the accurate classification of multi-eye diseases.

## 10. Conclusions

Millions of individuals worldwide are affected by diabetic retinopathy, hypertensive retinopathy, glaucoma, and contrast-related eye conditions. Preventing the development of these illnesses requires early identification. In this work, a fully automated system for detecting and classifying the four eye diseases is proposed. The model combines innovative preprocessing techniques with deep learning models to obtain the best possible results. The newly introduced Fluorescence Imaging Simulation image preprocessing algorithm is used to enhance the classification performance for these diseases. This novel image processing approach focuses on crucial areas in affected images, aiding in their identification. Additionally, we propose a feature fusion between the features extracted from state-of-the-art models (MobileNet, EfficientNet). The model has been extensively evaluated on four different pre-existing datasets as well as a newly created dataset that the authors assembled from other sources. Our suggested technique performs better than state-of-the-art models in the literature, according to a comparison analysis. An examination of their strengths and limitations proves the effectiveness of our approach over established models. To validate the efficacy of the proposed methodology, additional testing on a large, diversified dataset comprising a substantial number of possible sickness cases is required. Future research may explore the analysis of new datasets using NASNet or MobileNet, along with other augmentation techniques. This approach is not a decision support system, but rather a prescreening and automated illness detection tool. Future studies should concentrate on reducing the sensitivity to picture quality, in order to offer substantial flexibility and dependability across diverse healthcare settings.

## Figures and Tables

**Table 1:** A comparison of the present literature.

Reference	Methodology	Datasets	Models
[29]	Classification was made by convolutional neural network	MESSIDOR, DIARETDB, STARE	CNN
[30]	Preprocessing of image then passing it to a convolutional neural network to predict if the patient is diabetic	High-Resolution Fundus (HRF)	CNN
[31]	Gray level co-occurrence matrices (GLCMs), classifiers like Random Forests, Support Vector Machine (SVM), gradient boost, AdaBoost, Naïve Bayes, and Gaussian	MESSIDOR	GLCM
[35]	Disk-aware ensemble network	SCES, SINDI	CNN
[37]	Random Forest classification using different features such as the perimeter and area of the blood vessels and hemorrhages	STARE	Filters and Random Forests
[43]	CNN architecture	DiaretDB1	CNN
[44]	Deep belief network (DBN) and convolutional neural network (CNN)	DRIONS-DB, sjchoi86-HRF	CNN
[45]	Multiclass DED, auto-mated classification framework	MESSIDOR	CNN
[46]	Retinal illness classification based on selection utilizing Modified-Alexnet and ResNet-50 networks	-	AlexNet, ResNet50
[47]	DR severity detection and classification method based on enhanced pretrained NASNet Model	APTOS-2019, PAK-DR (Private)	NASNet
[48]	Using inceptionV3 model to detect and classify HR eye disease	PAK-HR (Private)	InceptionV3
[49]	A novel model is built based on the pretrained MobileNet architecture through the addition of dense blocks to make the network more efficient	PAK-HR (Private), DRIVE, DiaRetDB0	MobileNet

**Table 2:** Fluorescence Imaging Simulation.

Step	Explanation
Input Image Acquisition	Load the RGB image of the eye fundus from the dataset. All the images are normally captured with a high resolution and full color, for instance, 1125 × 1264 pixels.
Channel Separation	Separate the RGB image into its three color channels: red, green, and blue. Each channel is considered to be an individual gray-scale image representing the intensity of that color.
Green Channel Enhancement	Strengthen the green channel by scaling the intensity values with the formula G′ = *α* × G. Here, G is the source of the green channel, and *α* the scaling factor, which often takes a value of 2.0 to amplify the green’s intensity.
Image Reconstruction	Reconstruct the RGB image by combining the original red and blue with the enhanced green channel. I″ is given by I′ = merge (R, G′, B), where R and B are the original red and blue channels.
Blue Channel Suppression	This will turn off the blue channel to B = 0, which might allow the fluorescence effect to be more apparent because of the loss of the blue component but strongly showing the green fluorescence.
Colormap Application (Optional)	Just apply a colormap—for example, ‘HOT’—to emphasize the fluorescence more: it will just stretch the color palette of the image towards the warm end to show strongly bright green areas.
Output Image	Save or show the final processed image. This will shift the color scheme of the outputted image to accentuate green fluorescence and therefore pinpoint the areas of interest.

**Table 3:** Collected Datasets of Eye disorders used to train the CAD-EYE system.

Ref.	Datasets	Normal	Diabetic Retinopathy	Hypertensive Retinopathy	Glaucoma	Contrast	Total
[53]	Eyepacs, Aptos, Messidor	23,125	23,125	-	-	-	46,250
[54]	Eye disease dataset	-	-	-	50	100	150
[55]	Eye disease classification	250	250	-	250	250	1000
[56]	Dataset for different eye diseases	1637	-	-	1637	1637	4101
[57]	DiaRetDB1	100	100	-	-	-	200
Private	PAK-HR	3000	-	3000	-	-	6000
Private	DR-Insight	1000	-	4000	-	-	5000
Private	Imam-HR	1130	-	2040	-	-	3170
-	-	30,242	23,475	9040	1937	1987	65,871

**Table 4:** Auto Augmentation Algorithm steps.

Step	Number
1	Importing the necessary python libraries (albumentations, torchvision, and torch).
2	Write the code for the get_autoaugment_transform() function. The AutoAugment policy and other augmentation settings are set up by this function.
3	The main code will have the following functionalities: a. Load the dataset for augmentation. b. After the dataset has been loaded, apply the already defined AutoAugment transformation.
4	This step uses torch.utils.data to create the DataLoader class. During training, the shuffled batches of the augmented dataset will be generated by the DataLoader.
5	Define a basic CNN model class. With nn.Module, SimpleCNN is used. For the model’s training, configure the optimizer (like SGD or Adam) and loss function (like CrossEntropyLoss). Indicate the batch size and learning rate, among other hyperparameters, as well as the quantity of training epochs.
6	The following steps are repeated to train the model for a given number of epochs: a. Put the model in the training mode first. b. Call the DataLoader to generate batches of images from the augmented images along with their labels. c. Run the model in the forward pass mode. d. A defined loss function is used to compute the difference between reference labels and expected outputs. e. The computation of the model’s parameter gradients with respect to the loss is performed using a backward pass. f. Tweak the model’s parameters with the selected optimizer in step number one and use computed gradients. g. The model can be evaluated now on a separate validation dataset during training.
7	The last step is the evaluation of the model on a new dataset.

**Table 5:** Steps of the CAD-EYE feature map extraction Algorithm.

Step	Operation		Explanations	
1	Load trained Models	Pre-	Extract features from images using EfficientNetB0 and MobileNetV2.	
2	Freeze ers	Lay-	Freeze the models trained by weight and transform the models to non-trainable ones.	
3	Global Average Pooling		$X_{M}^{\mathrm{Pooled}\;}=\;\mathrm{Global}\;\mathrm{AveragePooling}\;2D(X_{M}^{frozen})$ Apply global average pooling to both MobileNetV2 features and EfficientNetB0 features.	
4	Concatenation		$X_{\mathrm{Concat}\;}=\;\mathrm{Concatenate}\;\left(X_{M}^{\mathrm{Poolea}\;},X_{E}^{\mathrm{Poolea}}\right)$ pooled features.	Concatenate
5	Dense ers	Lay-	*X_dense_*_1_ = *Dense*(128, *activation* = ‘*relu*’)(*X_Concat_*). Apply the dense layer with ReLU activation. *X_dense_*_2_ = *Dense*(*num_classes_*, *activation* = ‘*softmax*’)(*X_dense_*_1_). The final dense layer for classification.	
6	Fusion Model Definition		Combine the features extracted from MobileNetV2 and EfficientNet models to train the model.	
7	Compile the Model		Using the compile function, we compiled the model. The function parameters include the metrics used in evaluating (accuracy) and the optimizer (Adam).	
8	Train Model	the	Using the fit function, the model is trained for 10 epochs. In this instruction, we specify for the fit function the training, validation, and test data arrays.	

**Table 6:** Notation table.

Techniques for Augmentation	Values
B	batch
X	batch minimum activating value
*µ_B_*	mini-batch mean
$\sigma_{B}^{2}$	mini-batch variance
*ϵ*	constant added for numerical stability
*β*	learning parameter
*γ*	learning parameter

**Table 7:** Proposed XGBoost classifier algorithm.

Step	Description	Input		Output	
1	Initialize XGBoost model	-		XGBoost model with hyperparameters (*η*, *λ*, *τ*)	
2	Train XGBoost model on normal and abnormal samples	Training data: X = (*x*_1_, *a*_1_), (*x*_2_, *a*_2_), …, (*x_m_*, *a_m_*) Labels: a = 0, 1		Trained model	XGBoost
3	Use depth-wise Conv2D instead of Conv2D	Feature map x = *a*_1_, *a*_2_, *a*_3_, …, *a_n_*		Modified map	feature
	Build classifier based on XGBoost				
4	Train XGBoost model Generate ensemble of trees Training data: X, Labels: a	Trained XGBoost model and ensemble of decision trees		-	
5	Allocate class label for testing samples	*Testing data*: *x* *xtest*1, *xtest*2, *xtest*3, …, *xtestk*	=	Predicted class labels for *X_test_*	
6	Output identification of normal retinographic samples and hypertensive retinopathy (HR)	Predicted class labels		Recognition result for diabetic retinopathy, hypertensive retinopathy, glaucoma, and contrast samples	

**Table 8:** Comparison between different DL models using performance metrics.

Models	Sensitivity (SE)	Specificity (SP)	Accuracy	F1-Score
VGG16	68%	64%	69%	68.8%
VGG19	70.9%	70.8%	71.1%	71%
InceptionV3	73.2%	74.5%	75%	74.7%
ResNet50	83.5%	82%	80.2%	80.8%
Xception	81.7%	80.4%	81.2%	81%
MobileNet	89.5%	88.9%	89.4%	89%
DenseNet-169	89.9%	90%	90%	90%
EfficientNet	88%	79.8%	88%	88%
CAD-EYE	97.3%	97.9%	98%	98%

**Table 9:** Performance assessment of EDC Dataset.

Model	Dataset	Sensitivity (SE)	Specificity (SP)	F1-Score	Recall	Accuracy
CAD-EYE	EDC	99.50	99.68	99.95	99.98	1.0

**Table 10:** This table reveals that CAD-EYE outperforms EDC [58] with superior performance.

Dataset	Sensitivity (SE)	Specificity (SP)	F1-Score	Recall	Accuracy
EDC Model [58]	98.7	96.3	98.7	98.7	99.4
CAD EYE	99.5	99.68	99.95	99.98	1.0

## Data Availability

The data will be available upon request from the authors for research purposes.
